# Development and Validation of Survival Scores and the Assessment of Spatial Trends in End-Stage Kidney Disease Outcomes

**DOI:** 10.21203/rs.3.rs-6523746/v1

**Published:** 2025-05-27

**Authors:** Nathan Meyer, Hossein Moradi Rekabdarkolaee, Brandon M. Varilek, Surachat Ngorsuraches, Patti Brooks, Jerry Schrier, Semhar Michael

**Affiliations:** South Dakota State University; South Dakota State University; University of Nebraska Medical Center; Auburn University; Dakota State University; Avera McKennan Hospital & University Health Center, Sioux Falls; South Dakota State University

**Keywords:** ESKD, mixture cure model, prognostic model, risk score, USRDS, survival score

## Abstract

**Background:**

There is a need to create new mortality prediction models for end-stage kidney disease (ESKD). This study aimed to develop and validate survival scores for patients with ESKD using a mixture cure model (MCM) including assessing the spatial trends in ESKD outcomes.

**Methods:**

This study used a United States Renal Data System (USRDS) dataset that contains 2,228,693 people with incident ESKD from 2000 through 2020, including those on dialysis or had at least one transplant. Many variables, including demographic and comorbid factors, were included within an MCM. This MCM was used to develop seven survival scores that would be summarized geographically. These survival scores are shown using maps of the United States and validated using the clinical measurements found within the USRDS dataset.

**Results:**

Many spatial survival trends across the United States were observed that could be validated using the USRDS data and current literature. The Appalachian and Great Plains regions of the United States contained individuals who mostly had lower survivability. Conversely, individuals residing around Southern California, in the Southeast, and around the Texas-Mexico border had higher survivability. Most of these findings aligned with previous studies. Furthermore, many of the trends could be explained by both the coefficient estimates of the MCM and the characteristics of the individuals living in each region. For example, the MCM coefficient estimates found Hispanics to have a higher survivability than their non-Hispanic counterparts, which aligned with the predominantly Hispanic-populated area of the Texas-Mexico border. Lastly, serum creatine, a USRDS variable not used within the MCM, was found to have a moderately positive, linear relationship with the survival scores developed.

**Conclusions:**

The survival scores developed and validated may benefit practitioners and policy-makers in more effectively addressing ESKD disparities.

## Background

In 2022, an average of 360 people in the United States (U.S.) initiated treatment for end-stage kidney disease (ESKD) each day [1]. In the same year, the adjusted incidence rate for all-cause mortality in adult ESKD patients was approximately 146 deaths per 1,000 person-years. The U.S. Renal Data System (USRDS) 2024 Annual Data Report relays these findings and also shares the many vast differences for mortality as partitioned by modality, age, race, ethnicity, and other factors [1]. For example, a study points out that American Indian / Alaska Native (AI/AN) persons disproportionately suffer from ESKD when compared to non-Hispanic White persons [2, 3]. This study also states that this disparity is especially true in remote places, such as in the AI/AN tribal lands of South Dakota, where receiving a transplant, the treatment option of choice, becomes unachievable for many. Furthermore, ESKD patients face hard therapeutic decisions [4]. The consequences of their choice of medical care varies depending on the patient due to the heterogeneity seen across treatment outcomes [5]. Mortality risk scores for ESKD patients would aid in making treatment decisions [4]. A survival score would be the complement of a mortality risk score, which comes naturally from a survival model.

In ESKD, accurate prediction models for assessing patient risk are extremely important to develop and validate, as the risk scores created may improve both patient outcomes and clinical practice [6]. Mortality risk scores may also result in targeted population screening [7]. In other words, we may stratify the population (*e.g.*, low, medium, and high risk) such that the risk score of each person is automatically identified from patient profiles. This stratification of individual risk may also provide information on the complications for ESKD to help in prevention [8]. Further, using these risk scores within helpful tools may assist in prognosis, recognition of high-risk patients, and application of different therapeutics [9].

Existing mortality prediction models for ESKD patients have either concerns for applicability to the clinical setting or contain a risk of bias due to various reasons, including the study population selected.^3^ Hence, it was concluded that contemporary mortality prediction models still need to be developed and validated; external validation is essential to apply the mortality risk scores developed to a clinical setting [9]. In other words, data based on a different sample of individuals should be used to test the risk scores instead of using the same data used during model development [10]. Mortality risk scores have been developed for ESKD patients, but have focused on older patients [11, 12], patients in the early stages of receiving dialysis [13], or have developed other risk scores (*i.e.*, not mortality risk scores) that attempt to predict the risk of developing progressive chronic kidney disease [14] or kidney transplant failure [15].

Factors known to accelerate chronic kidney disease are also important for assessing ESKD mortality risk such as demographic variables (*e.g.*, age, sex, ethnicity), nephrotoxins (*e.g.*, smoking, alcohol, drugs), and medical factors (*e.g.*, cardiovascular disease, diabetes); furthermore, a large prospective study with considerable observational time may give more accurate (or useful) risk scores [7]. The survival scores we develop in this manuscript are based on a mixture cure model (MCM) developed using a large population of ESKD patients, allowing for a long follow-up time. This dataset also has many covariates, including the examples listed earlier. These survival scores are validated using not only these same variables but also variables not used when creating the survival scores. This validation step includes summarizing these survival scores geographically to better understand the underlying trends that emerge. Our primary objective is to develop and validate survival scores based on an MCM using the variables found within the USRDS dataset. In addition, these survival scores may be used to develop an easy-to-use tool that gives survival score predictions. This would enable both persons with ESKD and stakeholders, such as practitioners or policy-makers, to make data-informed decisions.

## Methods

### Dataset

The survival scores developed are based on a survival model fitted on a USRDS dataset [16, 17]. This dataset contains individuals with incident ESKD from 2000 through 2020 including both those who either are on dialysis or had at least one transplant. Time and event status were the outcome variables used in the survival analysis, such that the event of interest was all-cause mortality. Independent variables used within the model were age group, sex, race, Hispanic, primary disease, Liu comorbidity index, inability to ambulate, inability to transfer, needs assistance with daily activities, institutionalized, alcohol dependence, tobacco use, drug (illicit) dependence, amputation, toxic nephropathy, modality, transplant, employment, insurance, rurality, and region. The original dataset was reduced due to missing covariate values and some individuals that were over 108 years old. Furthermore, this study focuses on individuals within the 50 U.S. states and the District of Columbia. Because mortality for those on dialysis and those who receive a transplant widely differ [18], an MCM was used to capture this heterogeneity seen within the data.

### Mixture Cure Model Methodology

Let t be an observed time until the event of interest or time of right-censoring, x and z each be a vector of covariates, b and β each be a vector of coefficients, and $S_{0}\;(t)$ be the baseline uncured survival function. The MCM is then given as

S(t;\varvecx,\varvecz)=π(\varvecz)S(t;\varvecx)+1-π(\varvecz)

such that

$$\pi (\backslash varvecz)=\frac{e^{\backslash varvecb^{\backslash varvecT}\backslash varvecz}}{1\;+\;e^{\backslash varvecb^{\backslash varvecT}\backslash varvecz}}\;and\;S(t;\backslash varvecx)={\left(S_{0}(t)\right)}^{exp\left\{\backslash varvec\beta^{\backslash varvecT}\backslash varvecx\right\}}$$

represent the incidence (or the proportion of uncured individuals) and latency (or the uncured survival function), respectively [19]. The expectation-maximization algorithm is used to find estimates for b and β [20]. Further, bootstrap sampling is used to find standard errors for these parameter estimates [21]. An MCM was first fit to the USRDS dataset, and corresponding survival score summaries were extracted. Further details of applying an MCM to the USRDS dataset was previously presented [22].

### Concordance

The concordance statistic was used to compare different methods of fitting the MCM. Concordance attempts to estimate the probability of a prediction moving in the same direction as the observed values for any two observations [23]. The concordance statistic is useful in showing how well a given model may correctly order individual survival levels [24]. Let $y_{i}$ and $y_{j}$ be two observations with corresponding predictions ${\overset{ˆ}{y}}_{i}$ and ${\overset{ˆ}{y}}_{j}$ for i,j∈{1,…,n}. Then, the concordance statistic estimates $P\left({\hat{y}}_{i}>{\hat{y}}_{j}|y_{i}>y_{j}\right)$. In other words, it estimates the probability of a correct prediction given the actual values. Then,

$$C=\frac{c\;+\;t_{\overset{ˆ}{y}}/2}{c\;+\;d\;+\;t_{\overset{ˆ}{y}}}$$

is one possible estimate such that c,d and $t_{\overset{ˆ}{y}}$ represent the concordant, discordant, and tied observations within the predicted values, respectively [25]. A higher concordance value is thus considered better. A concordance value of 0.5 indicates the model may be thought of as being just as useful as flipping a coin when ordering individual risk levels. Lastly, a concordance value less than 0.5 would mean that the model is actively ordering individual risk levels in an incorrect manner (the model is worse than just flipping a coin when comparing individuals).

In a survival analysis setting, censoring must also be considered. The observed values, $y_{i}$’s, would be pairs of survival times and status indicators. Hence, censored times must be able to be compared against other times. For example, a censored time of two years would be considered larger than one year, whereas an observed time of three years is not necessarily greater than the censored time of two. Nor is the censored time of two years necessarily equal to another censored time of two. This is due to the assumption that censoring indicates to us only that the given individual has survived greater than the recorded time. Furthermore, ${\hat{y}}_{i}$’s would represent risks that are calculated by considering linear predictors. In other words, the multiplication of the vector of estimates by the observed covariate values. This means a concordant pair would then be represented by the observed value and predicted risk moving in opposite directions, whereas a discordant pair is indicated by the movement of the two in the same direction. For instance, an individual that has an observed time of two years and a risk of four and another person with an observed time of three years and a risk of one would be considered a concordant pair.

The infinitesimal jackknife method is used to find standard error estimates for each concordance estimate [25]. The ordinary jackknife method assumes $\overset{ˆ}{\theta }$ is an estimate for some parameter to be estimated, θ. Further, let ${\hat{\theta }}_{\left(i\right)}$ be the estimate when the $i^{\text{th}}$ observation is removed for i∈{1,...,n} [26]. Then,

$$var(\hat{\theta })=\frac{n\;-\;1}{n}\sum_{i=1}^{n}{\left({\hat{\theta }}_{\left(i\right)}-\overset{‾}{\theta }\right)}^{2}$$

such that $\overset{‾}{\theta }=\frac{1}{n}\sum_{i=1}^{n}{\hat{\theta }}_{\left(i\right)}$ is the sample mean. In context, the true concordance would be represented by θ. Note that this method does not require refitting the model using a new dataset each time; on the other hand, only the estimate for concordance is found each time based on the results of the model fit once. The infinitesimal jackknife method instead involves assigning weights to each observation. Hence, the ordinary jackknife method is equivalent to assigning a weight of one to all observations except the observation that is being left out is assigned a weight of zero. Instead, the infinitesimal jackknife method assigns weights close to zero to “leave out” an observation. This method is not discussed further. Instead, it is pointed out that the results for this method are extremely similar to the ordinary jackknife method for “moderate to large data” to which the USRDS dataset would fall under this category [25].

Another method of estimating the standard error is to fit each model multiple times using Monte Carlo cross-validation (MCCV). MCCV consists of partitioning the data into training and testing sets at random, calculating the desired statistic, and repeating these two steps multiple times. More specifically, each model is refit using the training set, and the estimated concordance of the testing set is found. Furthermore, partitioning the data randomly each time means that each partition is independent of all other partitions. In context, the concordance estimate is the desired statistic. Since we have survival data, each partition must have an adequate number of individuals that were not censored. Hence, each partition is created so that the event status remains proportional to the original dataset every time. The estimated standard error of the concordance is then equal to the standard deviation of each cross-validation sample divided by the square root of the sample size.

### Development of Survival Scores

The MCM is used to extract several survival scores at individual and geographic levels. Algorithm 1 shows an algorithm for the development of survival scores. After selecting the method for fitting the model, survival scores may be developed using both the USRDS dataset and the MCM results based on the chosen method. As indicated by the algorithm, these represent the input, whereas the output is the set of seven survival scores across counties. These seven survival scores mentioned are related to the survivability of an individual and are listed as follows:

Score 1 - probability of surviving beyond 2 years
Score 2 - probability of surviving beyond 5 years
Score 3 - probability of surviving beyond 10 years
Score 4 - probability of surviving beyond 15 years
Score 5 - time at 25% survival probability
Score 6 - time at 50% survival probability
Score 7 - time at 75% survival probability.

**Figure 1:**
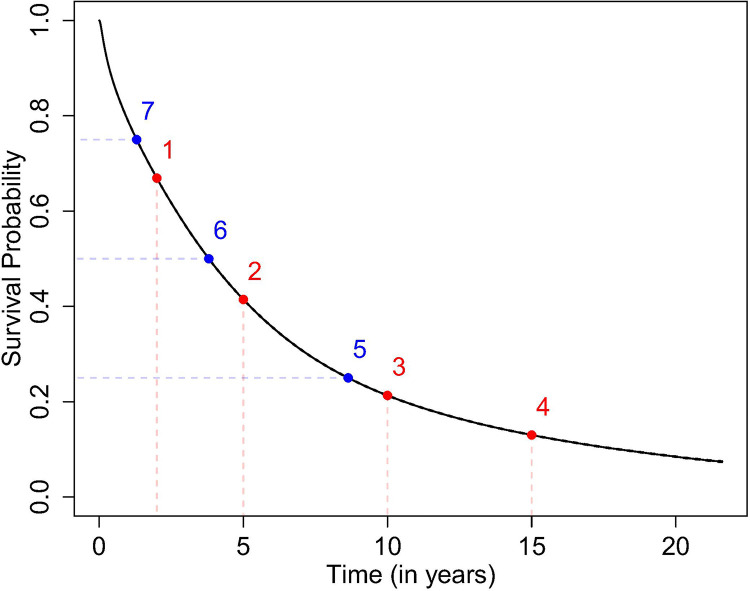
Kaplan-Meier survival plot of the USRDS dataset along with 95% confidence intervals given as black dashed-lines. Each labeled point represents one of the seven survival scores.

The last three survival scores represent the quartiles. The time variable within the dataset ranges from zero through 21 years with most events happening early on; hence, we used two, five, ten, and 15 years for the first four survival scores. Figure 1 displays a Kaplan-Meier survival curve along with the seven survival scores indicated as points on the line. This figure indicates that the survival scores adequately describe the entire survival curve. For example, the first four survival scores are somewhat equally spaced across the curve. The confidence intervals displayed are tight mostly due to the size of the dataset. Finally, the dataset must also be summarized by county when mapping these seven survival scores across the U.S. Three different possibilities of summarizing the data across counties is explored.

The first option is the univariate mode, which is the most intuitive and straightforward. The USRDS data is summarized across counties by finding the mode of each covariate used within the model. The seven different survival scores are then calculated using this summarized data and the MCM results. More specifically, the results of an MCM provide estimates for the coefficients involved within the model. This allows the survival function to be estimated using the values found for each of the covariates (*i.e.*, the mode of each county). This survival function then indicates the survival probability at any time. Note that survival probability represents the probability of surviving past a given time. Each of the seven survival scores may then be computed from this information giving the desired output of the algorithm.

The second case is the multivariate mode, which also considers finding a mode; however, the USRDS data is now summarized across counties by finding a simultaneous mode of multiple covariates. In particular, the most important predictors according to the MCM are used as the set of multiple covariates to find the multivariate mode of. To find the most important predictors, the magnitude of the z-values for each coefficient summed across covariate level (as there are two coefficients per covariate level in the MCM) is considered. A large z-value indicates the covariate has greater importance. An estimated cumulative distribution function may be considered to find a clearer cut-off for which predictors are most important. After this multivariate mode is calculated, the univariate mode of each of the remaining covariates is found as this information is needed to complete the calculation of the seven survival scores.

The third case given, the multiple mode, considers calculating the survival scores prior to summarizing the data. This contrasts with the first two cases, where the data was summarized first. Hence, the mode of the survival scores is found rather than of the covariates. Note that the mode is used, as opposed to the mean, due to the bimodal and highly right-skewed nature of the distribution of survival scores. In other words, the mean, unlike the mode, may not accurately represent this often highly skewed data. Since the survival scores are continuous, the estimated density was used to find the multiple mode. The word ‘multiple’ in the name refers to finding both the first and second mode (or the first and second peak of the distribution of survival scores). The second mode was considered as it may capture information about a different group of individuals. This gives two different sets of seven survival scores for this third case.

As indicated by Algorithm 1 for the third case, each of the seven survival scores are first calculated for all individuals within the USRDS dataset. Then, a kernel density estimation of each survival score across counties is found using the survival scores calculated. The default settings of the ‘density’ function in R was used which included using a normal kernel with the bandwidth following Silverman’s rule of thumb [27, 28]. Note that any of the methods for the development of survival scores could also be applied to the data at a different spatial level, such as zip codes. R Statistical Software version 4.3.2. was used for all statistical analyses [28].

## Results

This study included 2,228,693 people from the original 2,429,942 who had incident ESKD from 2000 through 2020 and met the inclusion criteria. The most common race was White among 85.1% of counties, followed by 12.9% for Black individuals, 1.7% for AIs/ANs, 0.2% for Asian individuals, and less than 0.1% for both Native Hawaiians / Pacific Islanders and the category of other races. In 96.5% of counties, the most common ethnicity was non-Hispanic leaving 3.5% for Hispanic individuals. Lastly, the most common primary disease was diabetes among 89.8% of counties, followed by 7.8% for hypertension, 1.8% for the category of other diseases, and 0.6% for glomerulonephritis / cystic kidney disease. The results section of this paper is structured as follows. First, model comparison is conducted using both an entire-based model and region-based models. This is followed by the determination of the most important predictors. Then, the application of the survival scores using maps of the United States is presented. Finally, model deployment is briefly discussed.

### Model Comparison

Prior to the application of survival scores, model comparison is conducted. We first considered the appropriateness of using one MCM versus multiple MCMs partitioned by geographical region. This would not only create models that better fit the data but also more accurate survival scores. The U.S. Department of Health and Human Services (HHS) partition the U.S. into ten different regions such that each state is assigned to only one region [29]. The concordance index was used to compare either fitting the MCM using the entire dataset versus fitting different MCMs to the ten regions separately. The Indian Health Service (IHS) Great Plains region that consists of North Dakota, South Dakota, Nebraska, and Iowa was considered in addition to HHS regions [30]. One difference with the full model is that the “region” covariate (which partitioned the U.S. into four regions) was not used when constructing MCMs partitioned by region. Finding the appropriate estimate of the concordance statistic for the MCM itself is not immediately intuitive [31], and the MCM is computationally expensive to fit due to the use of the expectation-maximization algorithm [32]. Hence, Cox regression models (a simplified survival analysis) were used instead to complete model comparison.

**Figure 2:**
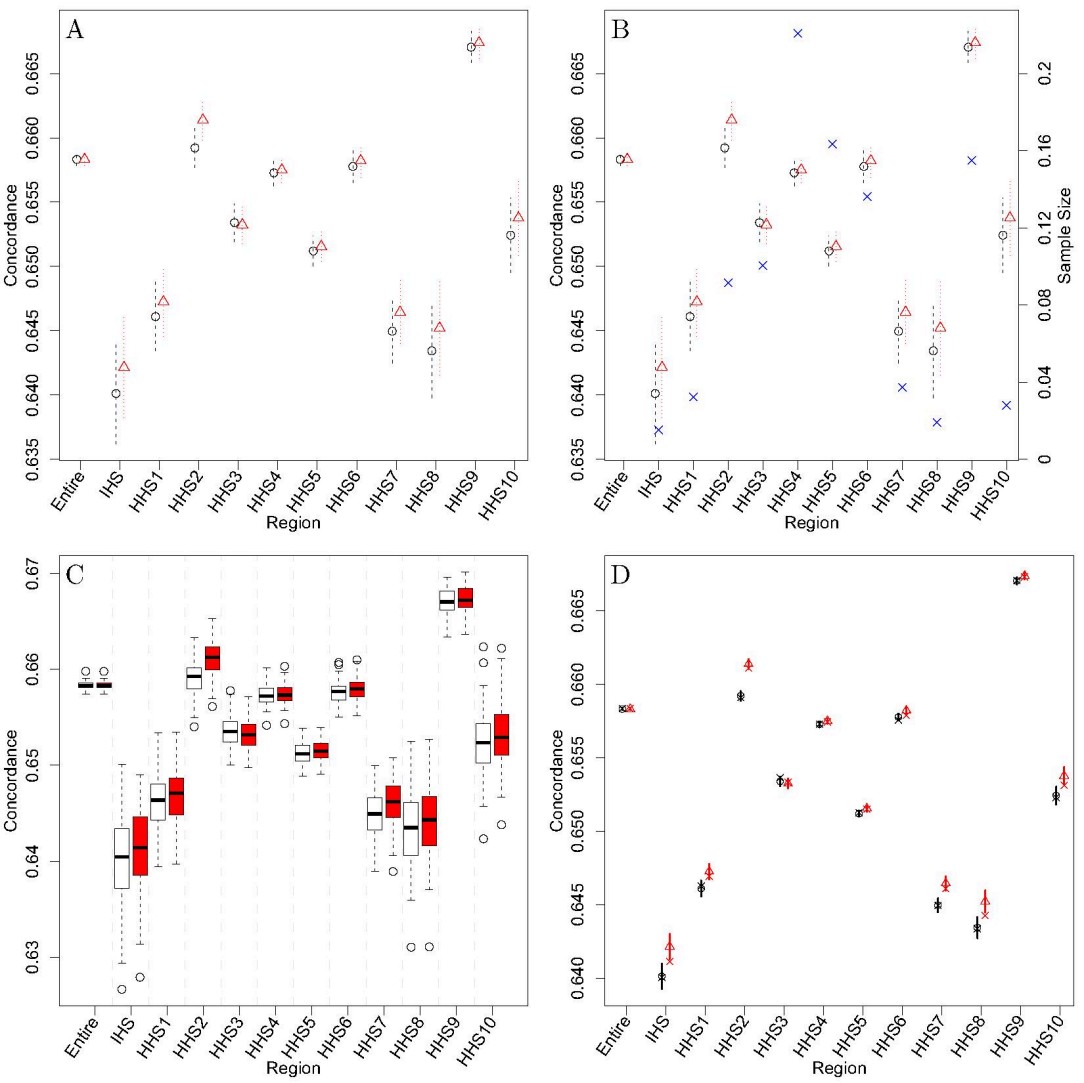
Concordance measures for either the entire-based model (black circles / white box plots) or the region-based models (red triangles / box plots). Subfigure (A) contains infinitesimal jackknife CIs; (B) displays a blue “X” to indicate the sample size of each region; (C) contains MCCV samples; (D) contains MCCV constructed CIs for the original concordance estimates with an “X” indicating the mean of the cross-validation sample.

The estimated concordance for each region and the corresponding 95% normal-based confidence intervals (CIs) using the infinitesimal jackknife method are shown in Fig. 2A. Figure 2B then shows the same information but with sample size in relation to the full dataset superimposed. Further, the concordance is shown to be the same for the ‘entire’ region since it is calculated using the same model either way. When performing MCCV, the training set consists of 80% of the data, whereas the testing set will be the remaining 20%. Figure 2C shows the results of 100 samples using MCCV. Further, Fig. 2D depicts how these MCCV samples are used to develop 95% normal-based CIs for the original concordance estimates.

Besides region three, the concordance estimates for the region-based models are consistently greater than the corresponding values for the entire-based model. This indicates that using the region-based models improves the ordering of individual survival levels and is thus a more accurate method. This conclusion about the Cox regression models will be applied to the MCMs. While the jackknife CIs in Fig. 2A overlap with one another when comparing the region- and entire-based models, some MCCV CIs in Fig. 2D do not and are much tighter. On the other hand, the CIs tend not to overlap across regions in both Figs. 2A and 2D. These differing concordance values across regions may be explained by the number of observations within each region. Figure 2B indicates that sample size differences among regions seem to be associated with concordance. Overall, the concordance indicates that the region-based models are slightly more accurate; thus, the results of the region-based MCMs were used in the development of the survival scores as opposed to the entire-based MCM results.

### Most Important Predictors

Using the previously described procedure for finding the most important predictors according to the MCM, the top 15 covariate factors with the highest importance values are listed:

**Table:** 

1. Transplant - At least one transplant	8. Institutionalized - Nursing home
2. Liu comorbidity index	9. Age group – 80 and older
3. Race - Black	10. Primary disease - Hypertension
4. Hispanic - Yes	11. Employment - Employed
5. Primary disease - Glomerulonephritis or Cystic kidney disease	12. Age group – 70 to 79
6. Primary disease - Other	13. Insurance - Medicare and Medicaid
7. Race - Asia	14. Race - Native Hawaiian / Pacific Islander
	15. Region - South

When fitting the MCM, indicator variables must be created for categorical variables. Thus, multiple z-values are found for some covariates (one z-value per indicator variable). Despite this limitation, the top five items with the highest value contained different covariates.

As expected, transplant status was the top variable listed. These top variables were used in further analyses to help indicate what covariates possibly influenced the survival scores. Furthermore, they were used as the subset of variables when considering the multivariate mode for summarizing the data across counties. However, finding the simultaneous mode of the top five covariates would be troublesome as no mode would almost always be found due to the inclusion of a continuous variable – the Liu comorbidity index. Thus, the Liu comorbidity index was not used when finding the simultaneous mode for the multivariate mode option. Further justification for using the top five predictor variables in further analyses is given in the supplemental materials.

### Survival Score Trends Across the United States

**Figure 3:**
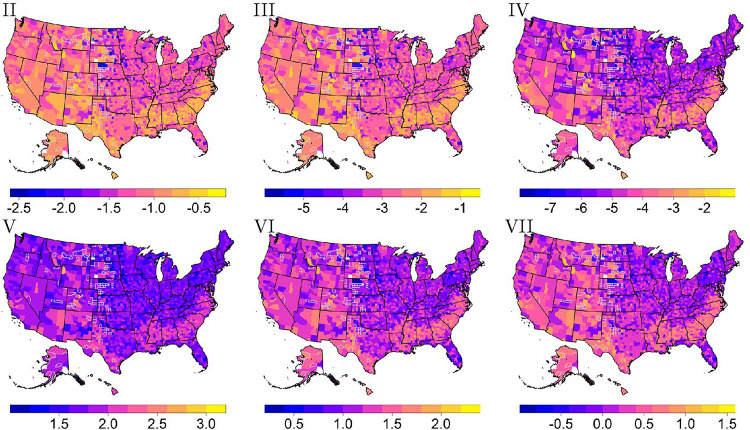
Survival scores two through seven calculated using the univariate mode (with a log transformation applied). Counties with gray borders indicate imputed values for suppressed data.

With the region-based MCMs and most important predictors selected, the survival scores may be mapped across the U.S. and validated. Figure 3 shows several maps of the U.S. when using the region-based MCMs to find each survival score across counties. In these maps, a larger survival score corresponds to a greater survivability. Also, the few counties that are colored white contain no persons within the USRDS dataset. Lastly, according to Centers for Medicare & Medicaid Services reporting rules, values representing one to ten individuals may not be reported or derived from reported work [33]. Thus, neighboring counties were considered to perform imputation when summarizing the dataset. More specifically, a nearest neighbors algorithm was used to perform the imputation where a mode or average, whichever is most appropriate, was found. The number of neighbors each county had varied; further, all neighbors for each county were used to complete the imputation process. Counties with imputed information have a gray border within the maps of the survival scores. Figure 3 shows similar trends for each survival score. For example, the Appalachian regions of the U.S. appear to have a lower survivability com- pared to surrounding areas. This is a faint trend seen according to any survival score, but it is more prominently shown within the map of the sixth survival score. A stronger conclusion is reached when considering the lower survivability found for individuals living in the Great Plains region of the U.S. (the area east of the Rocky Mountains). Alternatively, those living in the Southeast, around the Texas-Mexico border, and Southern California tend to have higher survivability.

**Figure 4:**
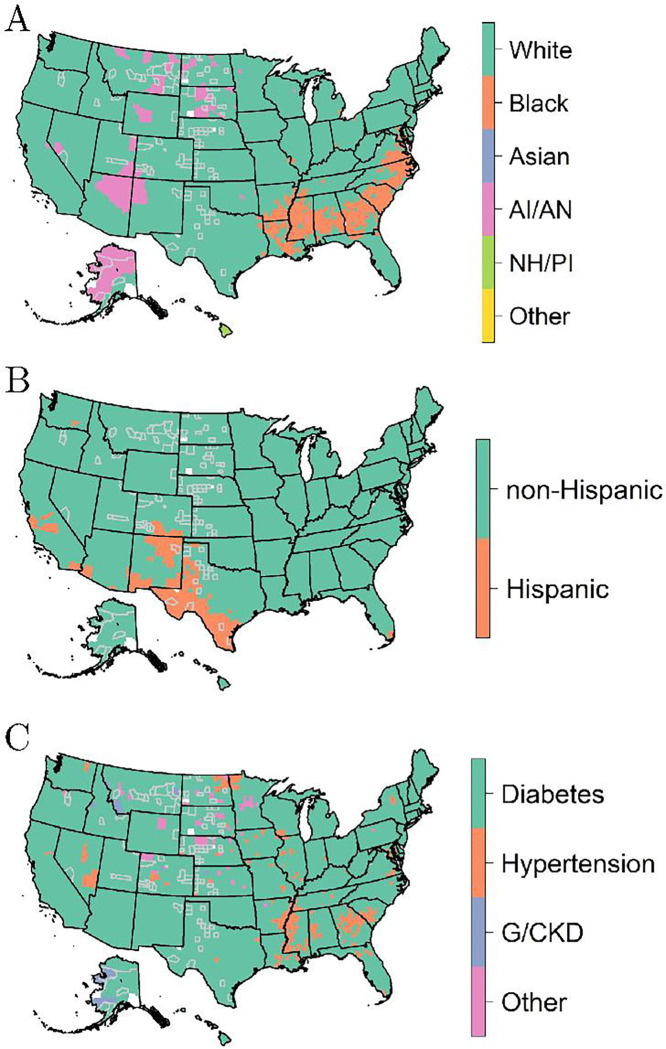
The mode of (A) race, (B) Hispanic status, and (C) primary disease across counties. Note that AI/AN is American Indian / Alaska Native, NH/PI is Native Hawaiian / Pacific Islander, and G/CKD is Glomerulonephritis / Cystic kidney disease. Counties with gray borders indicate imputed values for suppressed data.

Recall the top five most important predictors – transplant, Liu comorbidity index, race, Hispanic, and primary disease. These variables may help internally explain the survival scores shown across counties by investigating the trends within these variables across regions. Figures 4A through 4C display the most frequent race, Hispanic status, and primary disease across counties, respectively. These three variables appear to highly correlate with the trends seen within the resulting survival score maps from Fig. 3. For example, the characteristics of race and primary disease appear to be the main motivating factors for the trend seen in the Southeastern region of the U.S. More specifically, Black individuals and those with hypertension appear most frequently within the Southeast. This would indicate that these individuals have a higher survivability compared to White persons or those with diabetes when analyzing Figs. 3 and 4A. Further, the trend shown around the Texas-Mexico border seems to be greatly driven by Hispanic status. In other words, Figs. 3 and 4B show that Hispanic individuals appear to have a higher survivability based on the survival scores developed. Lastly, there appears to be a connection between locations in Figs. 4C where AIs/ANs appear most frequent (*i.e.*, AI/AN tribes) and places in Fig. 3 that indicate higher survivability. The other two most important variables previously mentioned were the Liu comorbidity index and transplant status. These maps showed nearly the same value each (*i.e.*, zero for the index and a mode of no transplant) across the entire U.S.

**Figure 5:**
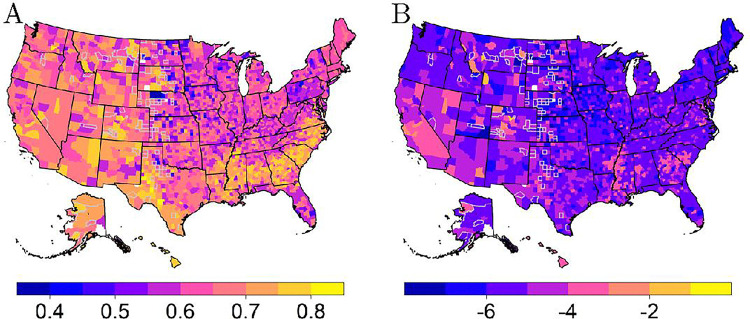
A comparison of the geographical results from the two parts of the MCM. Subfigure (A) uses only the latency portion of the MCM and displays survival score one whereas (B) displays the incidence with a logit transformation (the cure proportion is shown). Counties with gray borders indicate imputed values for suppressed data.

To further explain the trends seen, Fig. 5 visualizes the MCM split between its two parts. More specifically, the MCM has a latency portion that gives the survival probabilities for the individuals that are considered uncured. The MCM also has an incidence portion that gives the probability of being uncured for any individual. Note that the latency of the MCM relates to the survival function and thus may be summarized as discussed using the seven survival scores. Figure 5A displays only the first survival score. The map of the survival score for only the latency portion in Fig. 5A appears extremely similar to the maps of the survival scores given in Fig. 3. This implies that the survival scores of the MCM favors the latency portion. On the other hand, the map of the incidence in Fig. 5B shows a reduction in the number of individuals who have a higher probability of being cured within the aforementioned regions (*e.g.*, the Southeast). Furthermore, Table 1 shows the coefficient estimates from the entire-based MCM for the selected covariates of race, Hispanic, and primary disease. More specifically, the table shows the hazard and odds ratios which represent the latency and incidence portion of the model, respectively. The coefficient estimates given in this table further affirm the survival score trends discussed. See the supplemental materials for an extended version of Table 1 that includes all covariates. Overall, analyzing the profile maps of some variables used within the MCM and separating the two portions of the MCM appear to help validate the survival scores calculated using the univariate mode.

The second two cases for summarizing survival scores shown in Algorithm 1 may show further trends. As mentioned, the multivariate mode concerns finding a simultaneous mode of transplant, race, Hispanic, and primary disease across counties. A reduction in the number of variables for the multivariate mode is applied if no mode is found. For example, a multivariate mode of transplant, race, and Hispanic status (size three) is considered if a multivariate mode of size four (including primary disease) is not found. Note that the variables are removed in order of the most important variable list. When comparing the univariate and multivariate mode for each survival score across counties, high correlations range from about 80–96% across survival scores. In other words, the multivariate mode results in similar survival scores as the univariate mode. Most of the counties contained a multivariate mode of length four (the maximum length). A map of each survival score computed using the multivariate mode shows similar trends to each map of the univariate mode given in Fig. 3.

**Figure 6:**
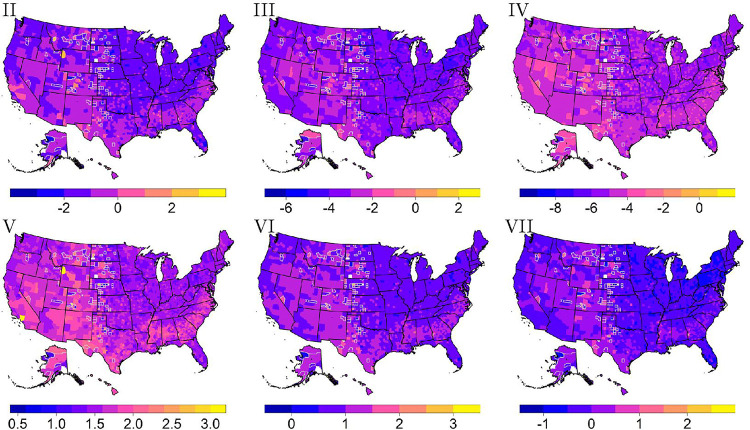
Survival scores two through four (with a logit transformation applied) and five through seven (with a log transformation applied) calculated using the first multiple-mode. Counties with gray borders indicate imputed values for suppressed data.

**Figure 7:**
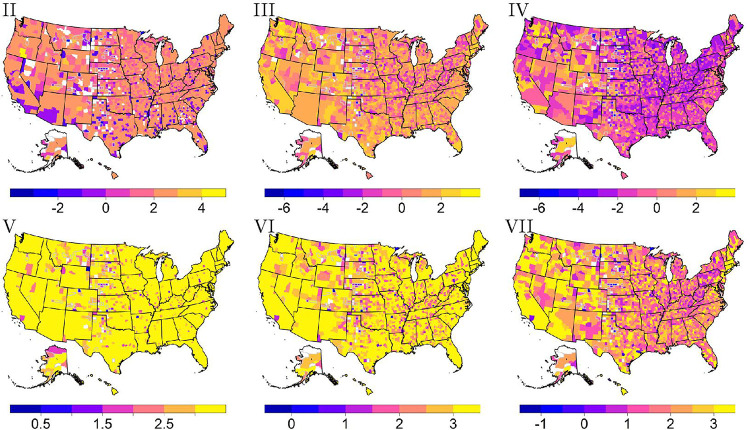
Survival scores two through four (with a logit transformation applied) and five through seven (with a log transformation applied) calculated using the second multiple-mode. Counties with gray borders indicate imputed values for suppressed data.

The multiple-mode method of summarizing survival scores includes finding either the first or second multiple-mode. The first multiple-mode is moderately to highly correlated with the univariate mode as correlations range from 65–72% across survival scores. As expected, a map of survival scores two through seven calculated using the first multiple-mode shown in Fig. 6 gives similar trends to each map of the univariate mode shown in Fig. 3. On the other hand, the second multiple-mode is uncorrelated with the univariate mode as correlations range from − 5–28% across survival scores. This lack of correlation is intuitive as the second multiple-mode focuses on a different group of individuals. As seen in Fig. 7, a map of the survival scores two through seven calculated using the second multiple-mode shows additional counties that are missing due to a second mode not able to be calculated from the density. Furthermore, Fig. 7 shows that survival scores five and six are mainly comprised of higher survival scores. Since the second mode often represents those that have a higher survivability, survival score maps five and six indicate that many of these individuals do not reach a 25% or 50% survival probability within the approximate 21.5 years of follow-up time. In other words, the largest time of about 21.5 is the closest time value to these survival probabilities. The seventh survival score map does indicate that more of these individuals do reach a 75% survival probability. Lastly, the second multiple-mode displayed in Fig. 7 does not show the same trends as the univariate mode pictured in Fig. 3 matching the correlation results aforementioned.

**Figure 8:**
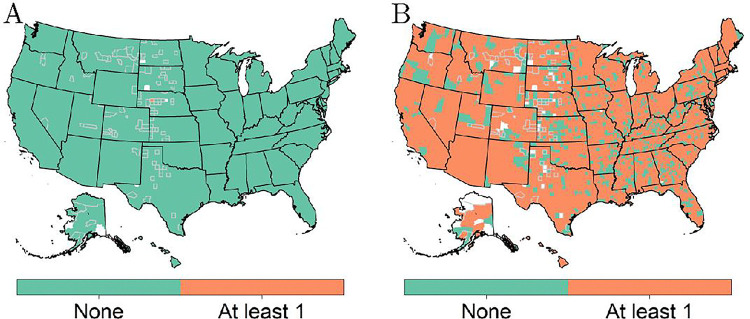
Transplant status for individuals within the (A) first mode and (B) second mode for survival score six. Counties with gray borders indicate imputed values for suppressed data.

The second mode will often be greater than the first mode due to the right-skewness of the data. This implies that the second mode should capture individuals who have a higher survivability. To help explain the differing trends within these two modes, we now consider the characteristics of those within the first mode group versus the second mode group. To find the characteristics, an individual is found with a similar survival score as reported for each county and for each mode. The characteristics of these individuals are then mapped across county. For example, Figs. 8A and 8B indicate that transplant status is the main motivating factor for the difference seen between the first and second mode. In other words, a person that aligns with the first mode is most likely without a transplant, whereas the second mode is mainly composed of individuals with a transplant.

Further clinical variables from the USRDS dataset may be used to further validate the survival scores found. The clinical variables include height; weight; body mass index (BMI); lipid profile TC, TG, HDL, and LDL; serum creatinine; blood urea nitrogen (BUN); Hemoglobin; and, Hemoglobin A1C. Outliers of these clinical variables were removed prior to further analyses. Each variable was then compared to each of the seven survival scores at the individual, zip code, and county levels (with both the mean and median of each clinical variable being considered individually when summarizing geographically). The correlation between each was calculated. From all clinical variables available in the dataset, serum creatinine had the highest correlation for all seven survival scores. The remaining variables had little to no correlation at the individual and zip code levels. More specifically, serum creatinine compared to the various survival scores across counties showed a moderately positive correlation ranging between 45–50% (*i.e.*, survivability increases as serum creatinine increases). See the supplemental materials for more details.

### Model Deployment with a User Interface

A shiny application was developed to provide an easy-to-use user interface for interacting with not only the USRDS dataset but also the results of the MCM [34]. This application contained three parts. First, interactive barplots that aid in describing the dataset were provided, which include the covariates used within the MCM. Different types of barplots may be explored including ones partitioned by transplant status. Second, individual survival curves, partitioned by transplant status, were given based on the selection of variables that were used to fit the MCM. Interpretations of these survival plots were also provided including the predicted probability of being cured for a person with the specific covariates selected. The survival scores discussed in this paper were also interpreted and shown on these survival curves. Lastly, selected survival score maps were presented and made interactive. More specifically, the univariate mode calculated across counties was used. This shiny application may be accessed online [35].

## Discussion

In this work, an MCM is fitted to a USRDS dataset to develop and validate survival scores for ESKD patients across the U.S. The survival scores were calculated from region-based MCMs as these models provided more accurate results according to the concordance statistic. Seven different survival scores were developed using three different methods of summarizing the data geographically. The simpler approach of the univariate mode appeared to be sufficient at summarizing the data. Survival scores varied according to individual characteristics, allowing interesting trends to emerge when the survival scores were mapped across the U.S. The most important predictors from the MCM aided in the exploration and validation of the survival scores. The top five most important predictors were transplant, Liu comorbidity index, race, Hispanic, and primary disease. These variables were used to explain some of the trends seen within the survival scores.

The Southeast section of the U.S. indicates higher survivability, which appears to follow the same region where there is a significant proportion of both Black individuals and persons with hypertension. This is intuitive since the model showed lower hazard for black individuals in the latency portion. Further, the model showed both lower hazard and odds of being uncured for persons with hypertension. Similarly, the Texas-Mexico border indicates higher survivability, which appears to follow the same area where there is a significant proportion of Hispanic individuals. Again, this is intuitive as the model showed both lower hazard and odds of being uncured for Hispanic individuals. These relationships related to race and ethnicity are further supported within literature as both Hispanic and Black individuals are often reported to have a lower risk of mortality, which this is usually attributed to their quick progression of kidney disease [36–39].

Lower survivability for individuals living in the Great Plains region of the U.S. may be attributed to both the presence of many agricultural communities and rural areas. For example, rural residents face significant hardships in receiving ESKD treatment, such as dialysis care, resulting in higher mortality rates [40, 41]. Furthermore, individuals have a higher risk of developing chronic kidney disease when living in agricultural communities throughout the U.S. due to many factors, including geo-environmental and argo-environmental influences [42]. Another study shares the connection between the risk of ESKD and pesticide exposure [43]. Furthermore, we found a lower survivability in ESKD patients around the Appalachian regions which aligns with the results of a recent study using a USRDS dataset to explore ESKD mortality [44]. This same study also found lower age-standardized mortality rates near Southern California, matching our results about higher survivability. Additionally, this research found that lower ESKD survivability among counties is significantly associated with a lower percentage of Black residents. This result corresponds to the race variable’s heavy impact on the Southeastern region.

The variables used within the model explain the survival scores developed. In addition, the clinical variable serum creatinine, which was not included in the model development, showed correlation with the survival scores. Hence, this provides some outside model validation. We found that serum creatinine had a linearly positive relationship with the survival scores developed, which was moderately strong when summarized across counties. Counterintuitively, this relationship indicates that a higher serum creatinine level indicates a lower mortality. This relationship has been found in a previous study that conducted a retrospective study of incident ESKD patients living in Maryland and Virginia [45]. This article provided a few explanations for why this may have happened. For example, serum creatinine level as an indication of overall health may supersede the application of this measure to specifically kidney function. More specifically, muscle mass and nutritional status, for instance, may confound the relationship between ESKD and serum creatinine.

## Conclusions

The main limitation of this study is that external validation is required for the developed survival scores to be generalized and used within the clinical setting [10]. This study did make use of variables outside the MCM covariates to validate the survival scores; however, these outside variables originated from the same USRDS dataset used to fit the MCM. Thus, different observations are required to externally validate the survival scores developed. Overall, this study developed and internally validated survival scores based on MCMs for patients with ESKD using a large USRDS dataset. The spatial trends shown may enable future stakeholders to make data-informed decisions.

## Figures and Tables

**Table 1:** Entire-based MCM coefficient estimates and standard errors of select variables (all results reported here were significant at a 0.05 level).

Characteristic	Latency	Incidence
	Estimate (s.e.)	Estimate (s.e.)
**Race**		
White	(ref)	
Black	−0.340 (0.002)	0.302 (0.016)
Asian	−0.399 (0.006)	−0.677 (0.030)
AI/AN	−0.234 (0.008)	0.467 (0.061)
NH/PI	−0.379 (0.010)	−0.315 (0.065)
**Hispanic**, yes	−0.344 (0.003)	−0.530 (0.018)
**Primary disease**		
Diabetes	(ref)	
Hypertension	−0.022 (0.002)	−1.299 (0.026)
G/CKD	−0.205 (0.004)	−1.309 (0.023)

Abbreviations: s.e., standard error; AI/AN, American Indian / Alaska Native; NH/PI, Native Hawaiian / Pacific Islander; G/CKD, Glomerulonephritis / Cystic kidney disease

## Data Availability

Access to USRDS data is limited to researchers and institutions with approved Data Use Agreements and will not be released. The relevant code used to generate the results presented within this paper will be posted to GitHub.

## Abbreviations

AI/AN: American Indian / Alaska Native
BMI: Body mass index
BUN: Blood urea nitrogen
CI: Confidence interval
ESKD: End-stage kidney disease
G/CKD: Glomerulonephritis / Cystic kidney disease
HHS: Health and Human Services
HIS: Indian Health Service
MCCV: Monte Carlo cross-validation
MCM: Mixture cure model
NH/PI: Native Hawaiian / Pacific Islander
s.e.: standard error
U.S.: United States
USRDS: United States Renal Data System.
